# Supplementary material for: Asymmetric Synthesis of Quaternary Hydantoins via a Palladium-Catalyzed Aza-Heck Cyclization
Source: J Am Chem Soc. 2025 Nov 14;147(49):44692–8. doi: 10.1021/jacs.5c16022 (PMC12703750; doi:10.1021/jacs.5c16022)
Supplement: Supplementary file 2 [file ja5c16022_si_002.zip › All NMR FID Files/12/12_AllNMR/2/pdata/1/email_EM04-024-1-F29-end_2_1.pdf]

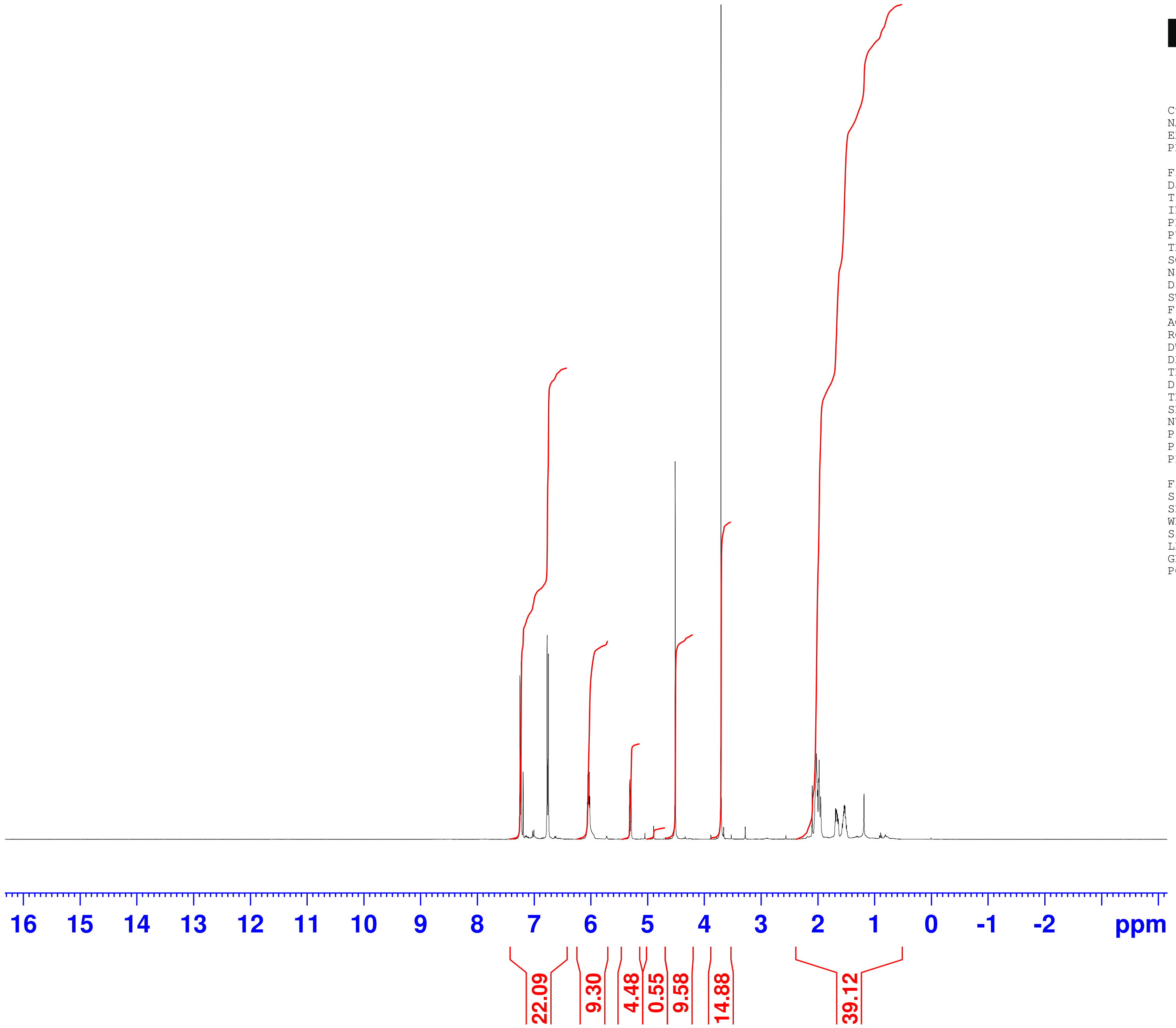


```
Current Data Parameters
NAME      EM04-024-1-F29-end
EXPNO                      2
PROCNO                     1

F2 - Acquisition Parameters
Date_               20230402
Time                   14.23 h
INSTRUM   Avance Neo 400 U of DE Brown Lab
PROBHD    Z824801_0171 (
PULPROG                 zg30
TD                     65536
SOLVENT                CDCl3
NS                        16
DS                         2
SWH                 8196.721 Hz
FIDRES              0.250144 Hz
AQ                 3.9976959 sec
RG                       101
DW                    61.000 usec
DE                     13.54 usec
TE                     298.2 K
D1                1.00000000 sec
TD0                        1
SFO1             400.1324708 MHz
NUC1                      1H
P0                      3.33 usec
P1                     10.00 usec
PLW1             16.44099998 W

F2 - Processing parameters
SI                     65536
SF               400.1300370 MHz
WDW                       EM
SSB                        0
LB                      0.30 Hz
GB                         0
PC                      1.00
```
